# Adaptation and validation of the Portuguese version of the Lithium Knowledge Test (LKT) of bipolar patients treated with lithium: cross-over study

**DOI:** 10.1186/1745-0179-2-34

**Published:** 2006-12-05

**Authors:** Adriane R Rosa, Ana Cristina Andreazza, Fernando Kratz Gazalle, Jose Sanchez-Moreno, Aida Santin, Airton Stein, Helena MT Barros, Eduard Vieta, Flávio Kapczinski

**Affiliations:** 1 Bipolar Disorders Program, Centro de Pesquisas, Clinic Hospital of Porto Alegre, Brazil and Post-Graduate Medical Science Program, Federal University of Rio Grande do Sul, Porto Alegre, RS, Brazil; 2 Department of Biochemistry, Science Institute of Basic Disease, Federal University of Rio Grande do Sul, Porto Alegre, RS, Brazil; 3 Post-Graduate Psychiatry Program, Federal University of Rio Grande do Sul, Porto Alegre, RS, Brazil; 4 Pharmacology Departament, Federal Fundation University of Medical Sciences of Porto Alegre, Brazil; 5 Bipolar Disorders Program, Clinical Institute of Neuroscience, Clinic Hospital of Barcelona, Villarroel 170, Barcelona 08036, Barcelona, Spain

## Abstract

**Objective:**

Adherence problems are a common feature among bipolar patients. A recent study showed that lithium knowledge was the main difference between adherent and non adherents bipolar patients. The Lithium Knowledge Test (LKT), a brief questionnaire, was developed as a means of identifying aspects of patients' practical and pharmacological knowledge which are important if therapy is to be safe and effective. The original English version is validated in psychiatric population, but a validated Portuguese one is not yet available.

**Methods:**

One hundred six patients selected were diagnosed with bipolar disorder (I or II) according to DSM-IV criteria and had to be on lithium treatment for at least one month. The LKT was administered on only one occasion. We analysed the internal consis tency, concurrent validity, sensitivity and specificity of the LKT for the detection of the knowledge about lithium treatment of bipolar patients.

**Results:**

The internal consistency, evaluated by Cronbach's alpha was 0.596. The mean of total score LKT by bipolar patients was 9.0 (SD: 0.75) for men and 8.74 (SD: 0.44) for women. Concurrent validity based on plasma lithium concentration showed a significant correlation between the total LKT score and plasma lithium (r = 0,232; p = 0.020). The sensitivity was 84% and specificity was 81%.

**Conclusion:**

LKT is a rapid, reliable instrument which appears to be as effective as a lengthier standard interview with a lithium clinic doctor, and which has a high level of acceptability to lithium patients. We found that the psychometric assessment of the Portuguese version of LKT showed good internal consistency, sensitivity and specificity.

## Background

To date, lithium is probably still the gold standard for the long term treatment of bipolar disorder, as shown in classical papers [1], newer studies [2] and a recent and thoughtful meta analysis [3]. However, a marked gap has been noted between the efficacy of lithium in clinical trials and its effectiveness in ordinary clinical practice [4,5], this difference being almost certainly due to poor treatment adherence [4,6,7]. A survey conducted in the United States showed that the average time of lithium intake was as start as 76 days [8].

Adherence problems are a common feature among bipolar patients. A recent study showed that lithium knowledge was the main difference between adherent and non-adherent bipolar patients [9]. Several studies have shown that psychoeducative interventions are associated with high adherence rates, stabilization of plasma lithium levels, reduction of the total number of episodes and total number of patients needing to hospitalization [10-12]. The fact that patients are informed about the disease, the treatment and the risks of not treating it positively, influences adherence, because it facilitates their acceptance of the disease and maintenance therapy [13-15].

The Lithium Knowledge Test (LKT) a brief questionnaire, was developed as a means of identifying aspects of patients' practical and pharmacological knowledge which are important if therapy is to be safe and effective. One point is scored for each correct answer and one is deducted for each wrong answer, giving a total Lithium Knowledge Score. Some incorrect answers, since they constitute potential hazards to patients on lithium, are added up to give a Lithium Hazard Score (LHS). The original English version has been validated in psychiatric population and has shown good reliability [16].

## Methods

### 2.1. Subjects

The study was conducted in two psychiatric outpatient services specialized in mood disorders in the city of Porto Alegre, Brazil. The psychiatric patients selected were diagnosed with bipolar disorder, I and II according to DSM-IV criteria. The study was approved by the Ethics Committees on Research of the hospital where the research took place, where the research took place and was carried out in compliance with the Helsinki Declaration.

The patient selection criteria included a well-established diagnosis, being on lithium treatment for at least one month, and regularly complying with the weekly visits scheduled in two psychiatric outpatients who agreed in take part in the survey.

In the weekly consultations, the patients were evaluated for both their symptoms and general state. The patients participated in psycho-educational groups about lithium with a specialized nurse and support groups with psychiatrists to discuss topics related to the disease.

The patients who agreed to participate in the study gave informed consent, were interviewed and had blood drawn immediately for comparison between the concentrations of lithium in the plasma with the LKT responses.

### 2.2. Variables

#### Demographical data

Every subject gave information about marital status, work, age, gender and education level.

#### Current clinical status

Bipolar patients were diagnosed using the Structured Clinical Interview for DSM-IV TR (SCID) and all patients were followed up prospectively using mood charts.

#### LKT

LKT is a brief questionnaire to identify aspects of patients' practical and pharmacological knowledge which are important if therapy is to be safe and effective. The LKT comprises 20 questions, which 1 point to be added for every correct answer and 1 point to be deducted for every wrong one. The correct answers are: 1-b; 2-b-c-f-h; 3-a-d-f; 4-a-b; 5-a-d; 6-c-e-f-g-i-k; 7-a-c-e. The total LKT score is obtained by adding together the responses to the 20 points. The mean LKT scores were close to 6. The mean LKT scores were > 6 indicating more knowledgeable. The LKT Hazard comprises of 3, 4, 6 and 7 questions for rating of identify aspects of intoxication symptoms about lithium. The total LKT Hazard score was obtained by adding together the responses to the 9 points. The mean LKT Hazard score was close to 4 or more indicating more hazard.

The linguistic adaptation of the LKT started with document in Portuguese obtained by a translation back translation method [17,18]. The items resulting in optimal word equivalence with the original text were analysed by the five of psychiatric investigators that agreed upon translation. Subsequently, bilingual people evaluated the degree of equivalence between the English original and the Portuguese version, as shown table 1. Finally, the comprehension of each item was assessed with a sample of 106 bipolar patients.

**Table 1:** Teste de Conhecimento do Lítio

Marque com um X as respostas que o Sr considerar correta.
1. Na sua opinião o LÍTIO age: (a) Como tranqüilizante; (b) Para prevenir mudanças de humor; (c) Como pílula para dormir; (d) Como tratamento para deficiência de LÍTIO
2. Quais os efeitos colaterais o LÍTIO pode causar? (a) Prisão de ventre (dificuldade de ir aos pés); (b) Mal-estar como enjôos; (c) Tremores; (d) Dor de cabeça; (e) Palpitações; (f) Urinar mais; (g) Insônia; (h) Aumento de peso
3. O que você deve tentar não fazer enquanto estiver tomando LÍTIO? (a) Suar excessivamente; (b) Tomar medicamento para tosse; (c) Climas muito frio; (d) Ficar grávida; (e) Fazer exercícios vigorosos; (f) Tomar diuréticos
4. Se você tivesse uma diarréia aguda e vômitos, o que você faria?(a) Ligaria para o seu médico; (b) Pararia de tomar LÍTIO imediatamente; (c) Iria para a cama e continuaria tomando LÍTIO regularmente; (d) Chamaria uma ambulância; (e) Aumentaria a dose de LÍTIO
5. Para que os exames de sangue regulares são necessários? (a) Para medir a quantidade de LÍTIO em seu sangue; (b) Para verificar se a doença é recorrente; (c) Para verificar se tem anemia; (d) Para testar o funcionamento da glândula tireóide
6. Quais das seguintes afirmações são verdadeiras? (a) O LÍTIO deve ser tomado exatamente no mesmo horário todos os dias (b) Deve-se tomar doses extras de LÍTIO se você se sente deprimido (c) Não deve-se tomar LÍTIO pela manhã quando for fazer exame de sangue (d) É normal não tomar algumas doses de LÍTIO se você se sente bem (e) O LÍTIO não é eficaz se o nível sangüíneo estiver muito baixo (f) O LÍTIO tem efeitos tóxicos se o nível sangüíneo estiver muito alto (g) É comum o LÍTIO ser receitado por vários anos (h) Parar de tomar o LÍTIO por completo geralmente leva a uma recaída (i) O LÍTIO tem sido experimentado e testado por muitos anos (j) O LÍTIO tem sido substituído por medicamentos modernos mais eficazes (k) Uma recaída durante o tratamento com LÍTIO não prova que ele não seja eficaz para o indivíduo
7. Quando você estiver tomando LÍTIO, o que deveria evitar na sua alimentação? (a) Dieta para emagrecimento; (b) Comer queijo; (c) Reduzir o consumo de sal; (d) Comida vegetariana (e) Beber álcool

The LKT was administered on one occasion, when venous blood to determine lithium concentration was collected from each patient. The time to application of LKT was 10 minutes. We analysed the internal consistency, concurrent validity in relation to plasma lithium concentration, sensitivity and specificity. Internal consistency was assessed with Cronbach's Alpha for the total scale.

Concurrent validity was studied considering plasma lithium concentration and the score obtained on the LKT by means of Pearson's correlation.

To study the sensitivity, specificity and positive and negative values of the LKT, we calculated the proportion of fully adherent bipolar patients by plasma lithium concentration and the proportion of non-adherent bipolar patients identified as such.

#### Lithium plasma concentration

The subjects were contacted seven days before the scheduled appointment and were instructed to be at the hospital early in the morning, without having taken their lithium dose in the morning because the lithium blood levels were to be measured, respecting a 12-hour interval between the last dose of lithium and the blood sampling. Venous blood was collected from each patient into Vacutainer tube containing edetic acid. The whole blood was then centrifuged 1600 × g for 10 minutes and the plasma removed by aspiration. A 1/20 dilution in water was made of 99 μl of plasma. Lithium concentrations were measured on the plasma dilutions by the indirect method, using an Instrumentation Laboratory CELM Flame Photometer. Assays were performed in duplicate [19].

### Statistical analysis

Statistical analysis was performed using SPSS for Windows – Version 12.0 (SPSS Inc., Chicago, IL, USa). The Kolmogorov-Smirnov test was used to compare an observed cumulative distribution to a theoretical cumulative distribution function. ANOVA test was used for compare LKT scores and plasma levels of lithium. Pearson's correlation coefficient was performed to examine the relationship between plasma lithium concentration and LKT scores. The binomial variables (clinical state and LKT scores) were compared using Chi-Square test. Validity was assessed with Cronbach's alpha.

## Results

The sample included 73 (68.9%) women, mean age 43.56 ± 9.83 and 33 (31.1%) men with a mean age of 41.61 ± 9.72. At the time when the present study was carried out, there were 77 (71.7%) euthymic, 12 (11.1%) manic and 17 (17.2%) depressed patients, according to the mood charts and physician is assessment. Habits showed 40 (37.7%) were smokers, 70 (66%) used coffee on a regular basis, and 68 (64.2%) had tea on a regular basis.

The answers to the LKT were obtained from 106 bipolar patients and the mean score on the LKT was 9.0 ± 0.75 for men and 8.74 ± 0.44 for woman and LKT HAZARD was 4.06 ± 0.19 for men and 3.96 ± 0.15 for woman. Table 2 describes the principal sociodemographic and clinical characteristics of the study sample.

**Table 2:** Demographics and clinical characteristics

Euthymic	71.7%	
Manic	11.1%	
Depressed	17.2%	
Smokers	37.7%	
Coffe	66%	
Tea	64.2%	
	**Men**	**Women**
	31.1%	68.9%
Age	41,61 ± 9,72	43,56 ± 9,83
Mean total LKT	9,0 ± 0,75	8,74 ± 0,44
Mean LKT Hazard	4,06 ± 0,19	3,96 ± 0,15

The internal consistency coefficient obtained to 43 items and the mean Cronbach's alpha of 0.596, for the total scale indicating that the items are sufficiently homogeneous.

Concurrent validity based on diagnosis according to plasma lithium concentration showed a significant correlation between the total LKT score and plasma lithium levels (r = 0.232; p = 0.020). The patients with higher LKT scores are more likely to have plasma levels within the therapeutic range.

We analysed the scale's discriminative capacity for bipolar patients by means of the knowledge about lithium treatment performance on sensitivity and specificity analysis. The sensitivity was 84% and specificity was 81%.

There were no significant differences between the total LKT scores among manic, depressive and euthymic patients (Pearson Chi-Square < .05). Date not shown.

We analysed the frequency of positive/negative answers on the LKT test by bipolar patients in comparison with standard answers offered by the original scale, as shown in table 3.

**Table 3:** Frequency of answers from TCL

Questions	% true answer	% false answer
1^a^	81.9	18.1
1b	64.8	35.2
1c	95.2	4.8
1d	77.1	22.9
2^a^	66.7	33.3
2b	41.9	58.1
2c	68.6	31.4
2d	61.9	38.1
2e	66.7	33.3
2f	67.6	32.4
2g	74.3	25.7
2h	83.8	16.2
53^a^	14.3	85.7
53b	91.4	8.6
53c	96.2	3.8
53d	43.8	56.2
53e	96.2	3.8
53f	37.1	62.9
54^a^	68.6	31.4
54b	12.4	87.6
54c	89.5	10.5
54d	93.3	6.7
54e	99.0	1.0
55^a^	89.5	10.5
55b	95.2	4.8
55c	96.2	3.8
55d	7.6	92.4
56a	20.0	80.0
56b	95.2	4.8
56c	82.9	17.1
56d	89.5	10.5
56e	52.4	47.6
56f	61.0	39.0
56g	79.0	21.0
56h	16.3	83.7
56i	75.2	24.8
56j	76.2	23.8
56k	73.3	26.7
57a	19.0	81.0
57b	89.5	10.5
57c	19.0	81.0
57d	95.2	4.8
57e	81.7	18.3

There was a significantly negative correlation between age and LKT scores (r = -0.2; p = 0.04). Age was positively correlated with LKT HAZARD scores (r = 0.366; p = 0.001)

## Discussion

Psychometric tests may be useful tools for the assessment of bipolar patients, not only in research protocols, but also in clinical practice [20]. LKT is a rapid, reliable instrument which appears to be as effective as a lengthier standard interview with a lithium clinic doctor, and which has a high level of acceptability to lithium patients. We found that the psychometric assessment of the Portuguese version of LKT showed good internal consistency, sensitivity and specificity. In Brazilian bipolar patients, the Portuguese version of LKT showed total scores which were similar of those described by *Dharmendra et al. *[21], in Scottish bipolar patients (total scores 9.6). Indeed, there was a significant negative correlation age and LKT scores and a significant positive correlation between LKT and Hazard scores, as shown in previous studies [21,22]. In the convergent analysis the total LKT scores were significantly correlated with plasma lithium levels, meaning that, the patients with higher LKT scores were more likely to have plasma levels within the therapeutic levels of lithium. Moreover, sensitivity and specificity tests showed good results, reinforcing the validation of Portuguese version of the LKT.

The LKT scale asked bipolar patients about lithium pharmacology aspects, such as, side effects, lithium interactions, risks of intoxication, food and lithium, necessity of measuring lithium levels, continuation of the lithium dosages and others [23]. Our study indicated that *Could not you to do while you was taken lithium? Which of these would be sensible actions if you developed acute diarrhoea and vomiting? Why the regular blood tests are necessary? Lithium must be taken at exactly the same times each day? Lithium is not effective if the blood level falls too low? Stopping lithium altogheter usually leads to a relapse? Which of the following changes in diet can cause problems with lithium? *were mainly difficulties reported by bipolar patients, because of this that patients with lower LKT scores are more uninformed about intoxication risks.

The identification of problems about lithium treatment and bipolar disorder with LKT could contribute to better understanding of the difficulties that bipolar patients have, and offer this specific information in the clinical consultations. Indeed, knowledge about prescribed medication tends to be poor in the ederly and these patients could have higher risks of intoxicacion with lithium than younger patients. While it is possible that patients who have been on lithium for a long time did not receive comprehensive information as did those who commenced treatment more recently and they required some reeducation about lithium for some time [21].

The knowledge level is a predictor of the adherence rates in bipolar patients in according recent reported [9]. In fact, *Colom et al. *[24] had reported that educated bipolar patients showed lower relapses rates, lower number and during hospitalizations. Psychoeducation is important to definitely mean more than providing information to patients and has shown its efficacy in stabilizing lithium plasma levels by improving pharmacological treatment adherence.

It is important to highlight several methodological issues. First, as with any study of treatment adherence we are hampered by the likelihood that the sample might have included a representative sample. Such methodological problems affect all studies in this field, as individuals who are non- adherent with lithium are also likely to fail to agree to participate or fail to adhere with research protocols. Second, we did not assess current symptom severity or comorbidity of bipolar disorder with other psychiatric or physical disorders. Third, this was a cross-over study, as we assessed knowledge about medication in only one occasion. As yet, there is no research on the stability or variability of such beliefs over time.

## Conclusion

In conclusion, the LKT is a brief questionnaire to evaluate the knowledge level in bipolar patients, identify aspects of patients' practical and pharmacological knowledge which are important if therapy is to be safe and effective. The LKT scale may now be used in Portuguese speaking populations as a direct measure of knowledge on lithium as a valid indirect measure of treatment adherence.
